# Supplementary figures and images for: Protective Effect of Green Tea Consumption on Colorectal Cancer Varies by Lifestyle Factors
Source: Nutrients. 2019 Nov 1;11(11):2612. doi: 10.3390/nu11112612 (PMC6893578; doi:10.3390/nu11112612)

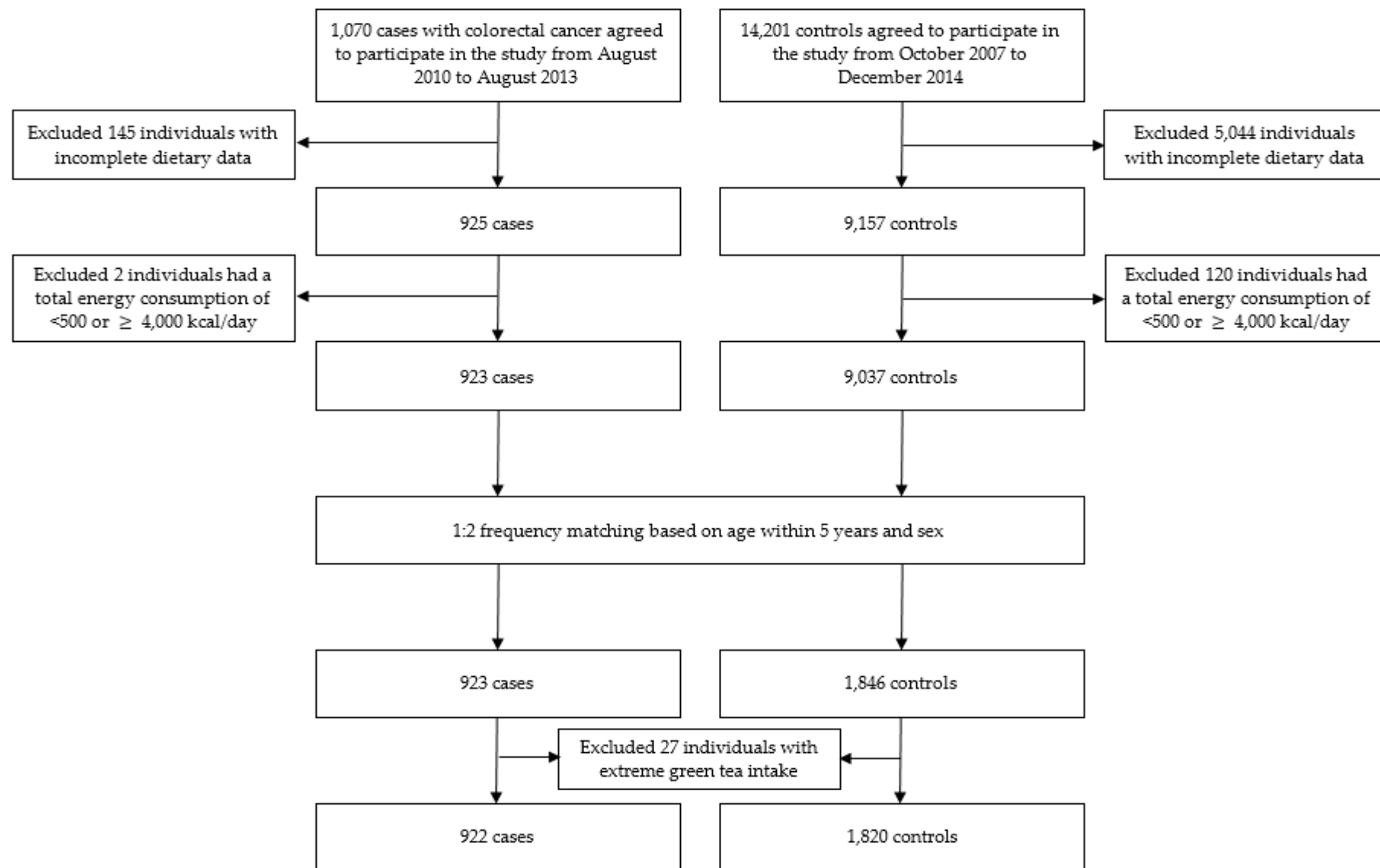

Figure S1. Flow chart of selection of the study population

Supplement: Supplementary file 1 [file nutrients-11-02612-s001.pdf]
